# Supplementary figures and images for: Impact of Bactrocera oleae on the fungal microbiota of ripe olive drupes
Source: PLoS One. 2018 Nov 29;13(11):e0199403. doi: 10.1371/journal.pone.0199403 (PMC6264826; doi:10.1371/journal.pone.0199403)

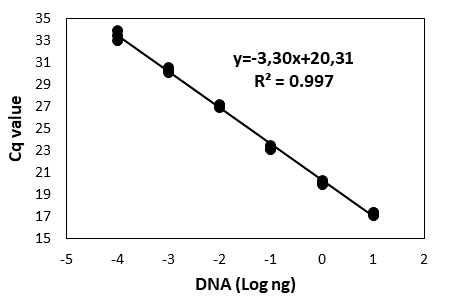

Supplement: S1 Fig — (PNG) [file pone.0199403.s001.png]
